# Neurological Damage by Coronaviruses: A Catastrophe in the Queue!

**DOI:** 10.3389/fimmu.2020.565521

**Published:** 2020-09-10

**Authors:** Ritu Mishra, Akhil C. Banerjea

**Affiliations:** Laboratory of Virology, National Institute of Immunology, New Delhi, India

**Keywords:** coronaviruses, SARS-CoV-2, Influenza, encephalitis, neuroinflammation, microglial priming, cytokine storm

## Abstract

Neurological disorders caused by neuroviral infections are an obvious pathogenic manifestation. However, non-neurotropic viruses or peripheral viral infections pose a considerable challenge as their neuropathological manifestations do not emerge because of primary infection. Their secondary or bystander pathologies develop much later, like a syndrome, during and after the recovery of patients from the primary disease. Massive inflammation caused by peripheral viral infections can trigger multiple neurological anomalies. These neurological damages may range from a general cognitive and motor dysfunction up to a wide spectrum of CNS anomalies, such as Acute Necrotizing Hemorrhagic Encephalopathy, Guillain-Barré syndrome, Encephalitis, Meningitis, anxiety, and other audio-visual disabilities. Peripheral viruses like Measles virus, Enteroviruses, Influenza viruses (HIN1 series), SARS-CoV-1, MERS-CoV, and, recently, SARS-CoV-2 are reported to cause various neurological manifestations in patients and are proven to be neuropathogenic even in cellular and animal model systems. This review presents a comprehensive picture of CNS susceptibilities toward these peripheral viral infections and explains some common underlying themes of their neuropathology in the human brain.

## Introduction

The common thread among herpes simplex virus (HSV), West Nile virus (WNV), enteroviruses like Poliovirus (PV), Coxsackievirus (CV), Influenza virus (IAV), Measles virus (MV), and especially human respiratory viruses like Coronaviruses (CoV) is that their primary site of infection is not the human Central Nervous System (CNS), yet they are all “Neuropathogenic” and exhibit fatal neurological abnormalities in humans. These viruses infect millions worldwide and cause a spectrum of neurological and psychiatric illnesses (1–3). If we look further into other chronic viral infections - such as Human Immunodeficiency Virus (HIV), John Cunningham Virus (JCV), Herpes Simplex Virus (HSV), Human Herpesvirus 6 (HHV-6), and Human T-lymphotropic virus (HTLV-1) – we realize that peripheral viral infections and the occurrence of neurological sequelae is quite a correlated phenomenon (4–6). Since neurological anomalies are fatal or potentially life-quality killers, it ultimately restricts the host survival and procreation activities. By this definition, invading the host nervous system seems a less promising evolutionary path (7). This makes the scenario of peripheral/respiratory viral invasion to CNS even more complicated.

The Central Nervous System (CNS) is strictly guarded by the blood-brain barrier (BBB) and effective immune surveillance. An intriguing question emerges over how these non-neurotropic viruses (most of them respiratory viruses) cause their respective primary pathology in the distal part of the host body, ultimately disrupting the sanctity of CNS. Is there a common pathway for all these viruses to enter CNS and cause neurological disorders or do they all use a unique route for causing their neuropathology?

In the usual course of infection, viruses usually cross the infected tissue and travel back to the bloodstream and lymph nodes and, through a hematogenous route, they can gain access to the CNS. Clinical observations show that any virus that is exposed to epithelial and endothelial linings usually breach the CNS because almost all human mucosal epithelial linings are heavily embedded with peripheral sensory nerves. However, in the case of non-neurotropic viral infections, it all seems opportunistic, because these viruses first exhibit their primary disease symptoms, followed by a secondary set of pathologies which include the neurological aberrations.

Backed up by over 100 years of extensive research and abundant literature, we can dig deeper into various viral strategies to disrupt CNS and make a wider comparison to find some commonly exploited pathways. In the following discussion, we will try to explore this issue on whether their entry into the CNS is opportunistic and accidental or whether these peripheral viruses are equipped to run such a multifaceted pathogenesis.

## Is It Easier for Respiratory Viruses to Enter the CNS?

**FIGURE 1:**
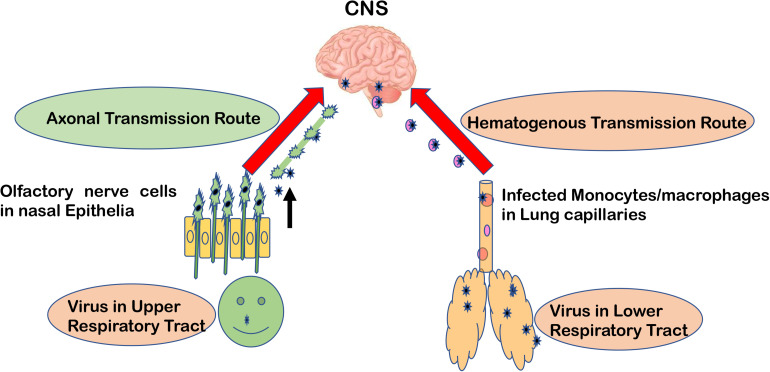
Major entry routes taken by viruses to reach the CNS. The schematic is describing two major transmission routes taken by viruses to reach the CNS: hematogenous transmission route and olfactory neuronal transport route. Respiratory viruses may infect the lower respiratory tract and lung epithelia, which are in close contact with fine blood capillaries for oxygen transport. Viruses travel toward the basolateral side of lung epithelia, enter the bloodstream, and eventually infect monocytes/macrophages in blood capillaries. As a “Trojan Horse” route, these infected monocytes can travel to the CNS. In the olfactory neuronal transmission route, viruses travel from one neuron to another via synaptic endings by using cell motor proteins to ultimately reach the CNS.

Major routes for entering the CNS are broadly categorized into two options. First, the hematogenous route, which means entry of peripheral infected blood cells like monocytes/macrophages as a “Trojan Horse” via crossing the BBB (schematic presentation as Figure 1). This route might also include infection of brain microvascular endothelial cells or the para-cellular passage via disrupting tight junctions and increasing brain endothelial permeability (8–11). Another major route of entry is the neuronal or axonal route, involving various peripheral nerves endings such as olfactory sensory neurons and intestinal nerve endings. It is called the axonal transport route because pathogens cross one neuron after another via synaptic nerve endings (8, 12, 13). Viruses are known to exploit the motor proteins, such as dynein and kinesin, for the retrograde and anterograde movement during neuronal transport (8, 9). Viruses can adapt both these routes to enter the CNS and these have been reviewed elsewhere in detail (12, 14, 15). A list of viruses has been provided as Table 1, showing their respective route of entry to the CNS.

**TABLE 1:** Multiple routes taken by viruses to enter CNS.

Virus name	Major route to enter CNS	References
Human Coronaviruses (HCoV)	Olfactory receptor neurons, Hematogenous route	PMID:16036791
		PMID:32167747
Influenza Virus (IAV)	Olfactory route	PMID:24550441
Respiratory syncytial Virus (RSV)	Olfactory receptor neurons, Olfactory bulb	PMID:31861926
Nipah Virus (NiV)	olfactory epithelium through the cribriform plate into the olfactory bulb	PMID:23071900
Herpes Simplex Virus (HSV)	Trigeminal ganglia, through vomeronasal system, and the hematogenous **route**	PMID:30863282
Rabies Virus	Transport through nerve endings.	PMID:2016778
Polio Virus (PV)	After oral ingestion, enteric nerve pathway, crossing of BBB.	PMID:22529845
Measles Virus (MV)	Crossing of BBB via infecting endothelial cells	PMID:27483301
West Nile Virus	Axonal transport	PMID:17939996
Japanese Encephalitis Virus (JEV)	Hematogenous route	PMID:25762733
Dengue Virus (DENV)	Hematogenous route	PMID:31293558
Hendra Virus	Direct Neuronal Infection	PMID:30985897

Olfactory receptor neurons display some key anatomical and functional features normally absent elsewhere. Olfactory receptor neurons are found predominantly in the upper respiratory tract, an area commonly known as the nasal cavity and nasal septum. When any odor gets dissolved in the mucosal lining of the nasal cavity, the apical part or dendrites of olfactory nerves sense it and transmit this signal to the basal part of olfactory nerves’ bodies for further transmission toward the brain (16). Olfactory receptor neuronal endings are directly exposed to the extrinsic stimuli and are capable of interacting enormously with all the environmental macromolecules. This interaction is responsible for our far-fetched ability to sense almost one trillion kinds of smells present in the universe (17). These olfactory nerve endings work in a chemoreceptor function. This means that any macromolecules can be physically incorporated inside olfactory nerve cells and travel in a *trans-*synaptic fashion to the CNS. This makes the olfactory nerve cells an extremely useful tool for respiratory viruses to reach the CNS without even bothering about the BBB barrier (18). Respiratory viruses, such as Influenza and Coronaviruses, are reported to preferably take this olfactory route for entering the CNS over other routes.

In the case of the current pandemic caused by SARS-CoV-2, the patients have been widely reported to exhibit the symptoms of anosmia/dysgeusia (i.e., smell and taste-related alterations) (19). The American Academy of Otolaryngology-Head and Neck Surgery (AAO-HNS) has also accepted anosmia and dysgeusia as the heralding sign for COVID-19 (20). The exact route taken by SARS-CoV-2 to enter the CNS has not yet been experimentally established. However, since this virus echoes almost a 79% similarity with previous SARS-CoV, we can expect some similarity in mode of entry to the CNS too. The presence of SARS-CoV (the cause of the 2009 and 2013 SARS epidemics) in the CNS has been well established, both in human patients and experimental animals. In the case of SARS-CoV, the brainstem region was reported to be heavily infected (21). SARS-CoV is also reported to spread through mechanoreceptors and chemoreceptors present in the lower respiratory tract to the medullary cardiorespiratory center of the brain via a synaptic route (22). This explains the widespread display of vascular and cardiac anomalies in severe patients of SARS-CoV epidemics. In the current SARS-CoV-2 pandemic too, patients are displaying intense vascular and cardiac anomalies which are becoming the main reason for death, however the exact cellular and molecular mechanisms still need to be experimentally validated (22).

## Respiratory Viruses Take the Hematogenous Route Too

Dijkman et al. reported that human coronaviruses (HCoV) cause far fewer cytopathic effects on primary human respiratory epithelial cells (23). They also showed that all HCoV strains were budding preferentially from the apical side of epithelial cells, but many viruses were also getting released from the basolateral side (23). This way they can pass through the epithelial barrier and enter the bloodstream and lymph nodes (Figure 1). This explains how they can infect leukocytes and travel via the hematogenous route to other distant organs like the CNS, kidney, and intestines, etc (1). This route has been shown to be taken by various other viruses like Measles virus (24), Nipah virus (25), and Influenza B virus (26, 27). Still, for SARS-CoV-2 infection, the preferred route of entry (either hematogenous or olfactory nerve transport) to the CNS needs more experimental validation.

## For Non-Neurotropic Viruses, Time Is Really an Illusion

By classical definitions, a pathogen and their pathogenesis represent a typical cause-and-effect relationship, described as “germ theory.” It was a gold standard established by Robert Koch in 1890, also known as the Koch’s Postulates. However, viruses seem to easily defy Koch’s Postulates when causing neurological disorders. In the case of viral infections, we have multiple examples where neurological sequelae of viral infections happened decades after the primary infection, when all the signature of viral genome, proteins, and toxins are long gone from the host. This gives us a serious challenge in assigning and linking the viral infection and resulting neuropathogenesis. Viruses are known to be an original root cause of several neurological syndromes either directly or indirectly. The difference between neuropathology caused by direct viral infection and that caused by potential bystander factors is still a highly explorable field.

For the sake of gaining perspective, if we go back to the history of the 1918 “Spanish Flu,” contemporary doctors and researchers observed an intriguing association of *encephalitis lethargica* (a debilitating nervous disorder in the form of sleep disorder, lethargy, and Parkinson-like symptoms) in flu patients after the recovery. These neurological perturbations could reduce the quality of a patient’s life for decades (3, 28). Following that, many episodes of the Influenza epidemic and their association with subsequent neurological disorders started convincing researchers that non-neurotropic/peripheral viruses, especially respiratory viruses, can affect the human brain with long-lasting neurological disorders (2). Those viral infections, where upon a strong immune response causes the brain to be cleared from viral particle through apoptosis and clearance of infected neurons, presented more challenging scenarios. For example, in Vesicular Stomatitis Virus (VSV) infection, which infects the brain serotonergic system, immune clearance results in a permanent loss of serotonin neurons, resulting in neurochemical and behavioral alterations (29). Lifelong neurological and psychiatric changes are brought upon by initial viral infection, however, no diagnosis could prove their association with a particular viral infection since the virus has long been eliminated long from the host.

Post-Polio syndrome presents a perfect example of how some viruses could remain latent in the brain and cause neurological dysfunction much later in life. Many cases have been reported where symptoms of poliomyelitis, such as weakness of affected limbs, came back after decades, sometimes even after 30–40 years (30, 31). Similarly, Chicken pox, caused by a double-strand DNA, enveloped herpes virus, remains latent inside neurons for decades (32, 33). It manifests as shingles when the active virus is produced from its latent genome and starts showing symptoms of itching and pain. It can produce a replication-competent virus which can travel down the axonal network and again infect the CNS neurons (33). In such a situation, we are faced with a challenge that diagnostic assays can detect the virus only in the blood or CSF, giving us a false diagnosis of patient being virus-free, while parenchymal tissues might still harbor the virus.

## Current Pandemic of COVID-19/SARS-CoV-2

Information on the general characteristics of SARS-CoV-2 virus, ACE2 receptor utilization, their transmission, and pathology is extensively available in the current flood of literature (34–38). Therefore, we will mainly focus on their neuropathological aspects and other unique features.

The initial evidences for SARS-CoV-2-related CNS damage emerged when a SARS-CoV-2 infected patient was diagnosed with acute necrotizing hemorrhagic encephalopathy along with other usual symptoms of COVID-19. Brain MRI images showed hemorrhagic rims in various cerebral segments such as bilateral thalami, medial temporal lobes, and sub-insular regions (39). However, the direct presence of SARS-CoV-2 viral particles within the brain and CSF still needed experimental validation which could further confirm their neurotropism (39). A strong association of SARS-CoV-2 with neurological disorders has been extensively discussed in the current literature. The latest updates on neuropathologies during COVID-19/SARS-CoV-2 can be explored in detail in the following references (40–42). Since this pandemic is still going on and most of the patients’ data collected so far are only from the acute respiratory illness phase, there is a lack of any documentation on the long-term impact of SARS-CoV-2 on the human CNS. The majority of data regarding neurological damage during SARS-CoV-2 infection are still without laboratory validation; so, we will have to look on how the previous episode of coronaviruses have influenced the CNS.

## Neuroinvasiveness of Coronaviruses

The presence of Coronavirus RNA (six previously known coronaviruses) have been reported in human brain autopsy samples which clearly established that, being primarily a respiratory virus, it is naturally neuroinvasive in human hosts and can successfully replicate within the brain (43). Further, it was established by many laboratories that a persistent infection of coronavirus happens in human CNS cells, such as oligodendrocytes and neuroglial cell lines (44–46). In a mouse model, HCoV-OC43 RNA were detected for much longer after the mice survived the acute encephalitis episodes due to coronavirus. HCoV-OC43 infection in a mice model was suggestive of a loss of hippocampal neurons (47). These reports suggest that coronavirus is not just a flu virus with harmless disease outcomes but is also able to maintain a persistent infection in CNS. Coronavirus infection might actually be a factor or co-factor for long-term neurological abnormalities to be revealed later in life, which we might not easily link with previous episodes of coronavirus infection.

Other strains of coronavirus, such as HCoV-229E, also infects human primary monocytes and activates them (48). These HCoV-229E-infected monocytes would turn into macrophages after the activation and can enter the CNS, particularly in immune-compromised patients. In mice models, it was observed that HCoV-229E could invade the CNS by taking advantage of an immune-suppressed environment (49). SARS-CoV-1, the causal factor of the previous SARS epidemic, was reported to infect monocytes to activate them into macrophages and dendritic cells (50, 51). These reports suggest that coronaviruses have developed a mechanism to take the hematogenous route for entering the CNS and these peripheral monocyte-macrophage populations can serve as a reservoir to maintain their replication within the CNS. There are also reports of coronavirus infection in brain endothelial cells shown in cell culture models (52). SARS-CoV-1 was reported to infect human brain endothelial cells (53). There are a few examples where the hematogenous route for entering the CNS is exemplified, however, coronaviruses are equally known to take the neuronal transmission route.

In the neuronal transmission route, the virus typically infects the neurons located peripherally and actively transports itself using the cellular actin-myosin machinery of neurons to enter the CNS (54). This route was confirmed in animal studies too, where coronaviruses such as HCoV-OC43 and SARS-CoV-1 were given to mice via intranasal injections. First, viruses were found in the respiratory tract and later were detected in the CNS of susceptible mice, confirming the olfactory route being taken to reach the CNS (55–58). Similarly, murine coronavirus (Mu-CoV) also takes the olfactory nerve route to enter the CNS (59). The HCoV-OC43 strain is experimentally shown to travel further from the olfactory bulb to other neural regions such as the cortex, hippocampus, and even the brainstem and spinal cord (39). This olfactory route being used by other viruses, such as influenza virus, Borna disease virus, and herpes simplex virus, has been extensively reviewed by Mori and colleagues (18).

## Coronavirus-Mediated Neuropathogenesis

Speculations about viral etiology behind common neurological diseases such as Parkinson’s disease (PD), ADEM (Acute disseminated encephalomyelitis), and multiple sclerosis (MS) has gained much strength in recent times. Particularly with various coronaviruses, such as HCoV-229E and HCoV-OC43, being detected within Parkinson’s disease (PD), ADEM (Acute disseminated encephalomyelitis), and multiple sclerosis (MS) patient’s brains, it is now discussed as a link between these neurodegenerative diseases and viral infections (43, 60). Even murine coronaviruses, known for demyelination in mice, have been found to contribute to oxidative tissue injury in human MS disease (61). This provided a very interesting, although worrisome, correlation that long-term infections with coronaviruses in human CNS may contribute toward MS-like lesions.

During coronavirus replication and proliferation in lung epithelial cells, the alveolar gas exchange phenomenon is severely hampered because of damaged lung epithelial cells (62). Afterward, the lack of oxygen in the entire body, including the CNS, may cause hypoxia disorders. It might activate anaerobic metabolic pathways and mitochondrial pathways within the brain (63, 64). The resultant acidosis in the brain can cause multiple dysfunctions like cerebral vasodilation, brain swellings, interstitial edema, headaches, and congestion, etc (65, 66). In severe cases it can lead to degraded brain functions, drowsiness, bulbar conjunctival edema, and eventually coma (67). Since patient’s reports from China and Italy have consistently shown that critical SARS-CoV-2 patients often develop severe hypoxia, subsequent cerebral damage is likely to happen.

This could be true for persistent infections of other coronaviruses within the CNS, however, more experimental evidence is needed to establish such a correlation for all the recent coronaviruses, such as SARS-CoV-1, MERS-CoV, and SARS-CoV-2.

## Why the Brain Is “The Most Susceptible” for Cytokine Storm?

Cytokines were once given the analogy of rain. As moderate and timely rain is needed to sustain life on earth, similarly a baseline amount of cytokines are actually needed for multiple cellular and physiological processes during the life of an organism (68, 69). Cytokine storm is a condition where the regulators of inflammatory immune responses, and thereby the production of cytokines, becomes out of proportion and out of place. This results in the production of an uncontrolled amount of inflammatory molecules. Cytokine storm happens in multiple bacterial and viral infections and septic conditions, however, the term “Cytokine storm” only gained its popularity after being discussed in the context of Influenza disease in 2005 (70).

**FIGURE 2:**
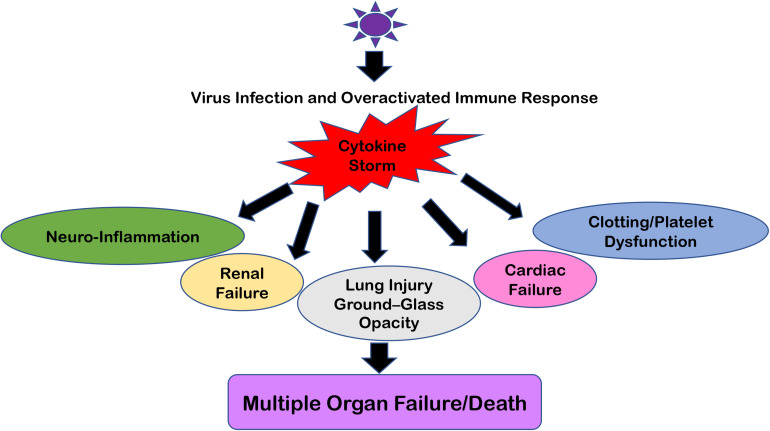
Multiple Organ Failure because of “Cytokine Storm.” The cartoon representation of how “Cytokine Storm” generated during respiratory viral infections can damage not only its primary infection site (i.e., lungs) but also disrupt the homeostasis at the kidneys, heart, intestine, cerebral parenchyma, and blood vessels because of the ubiquitous presence of ACE2 receptors. In severe cases, this leads to Multiple Organ Failure and the eventual death of patients.

This overreacting innate immune response creates the situation of “Cytokine Storm” which typically means that pro-inflammatory and anti-inflammatory cytokine levels are high in the serum of patients. These cytokine flares are usually destructive for all vital organs such as the heart, kidneys, and lungs (Figure 2). If such a scenario happens in the brain, this becomes extremely devastating and further paves the way for meningitis, encephalitis, meningoencephalitis, and overall neurodegenerative conditions (68, 71). Earlier, many viral infections of HIV-1, Dengue, and other flaviviruses have been reported to cause a cytokine storm phenomenon in the peripheral body which can reach the CNS via either disrupting the BBB or sometime even via *trans-*cellular crossing of the BBB (11, 72). The role of exosomes and other extracellular vesicles in Dengue viral hemorrhagic fever has also been discussed to highlight the importance of exosomal secretion in cytokine storm phenomenon (59). Even viral proteins such as HIV-1 Tat, Nef, and Dengue NS1 circulate in the bloodstream and trigger the generation and transportation of cytokines to multiple organs, including the CNS (73–77). Ultimately, all these influxes of peripheral inflammatory molecules inside the CNS activate the brain resident macrophages, i.e., microglia, which becomes hyper-activated and starts producing its own set of inflammatory molecules and causes neuroinflammation. This whole process of making microglia ready to act is known as “microglial priming.”

## “Microglial Priming” Might Explain a Lot

It is noteworthy that most of the chronic neurodegenerative diseases have an association with unresolved inflammation, also termed as neuroinflammation. These diseases are mainly Alzheimer’s disease (AD), Multiple sclerosis (MS), Parkinson’s Disease (PD), Huntington’s Disease (HD), ischemia, and strokes, (67, 68). Many researchers have explained the phenomenon of “Microglial Priming,” where microglia undergo multiplication followed by activation and stay in this “primed” condition for a very long time (78). This “priming” makes the microglia extremely susceptible toward a secondary inflammatory stimulus. Even a small molecular trigger can induce an exaggerated inflammatory response from the primed microglia (78, 79). It was experimentally validated that TNFa and the activation of microglia has a positive feedback loop relation (79). This explains why the brain is so susceptible to peripheral cytokine storm created by any route, be it bacterial, viral, or any other aging-related inflammatory abnormalities like rheumatoid arthritis. Cytokines produced by a ‘primed microglia’ pose a much higher threat in disrupting the homeostasis of the CNS than systemic inflammatory molecules added from peripheral monocytes (68, 69).

The secondary stimulus which activates microglia can begin in the CNS by many means, such as any neuroviral infections or bacterial invasion. However, it has been observed that conditions which contribute to systemic inflammation such as diabetes, ischemic conditions, and arthritis, are a major cause of priming microglia, and this can be particularly seen in elderly populations (80–82). This puts the elderly population at a much higher risk of experiencing neurological and cognitive disabilities if exposed to respiratory and/or other peripheral viral infections.

Other than microglia, astrocytes and neurons can also produce multiple cytokines upon exposure to external stimuli (69, 78). Such alterations in cerebral homeostasis mainly happens at synapses (78, 83–85). Synaptic anomalies are responsible for deficient learning and memory and disruption of normal behavior (83–85). This bridging of systemic inflammation with the CNS anomalies is a challenging interaction, however, at the same time it gives us a hopeful window to treat neurodegenerative conditions just by controlling systemic inflammation.

We will now briefly list the neurological anomalies caused by the following viral invaders.

## SARS-CoV

SARS-CoV-1 was the reason for the 2003, 2009, and 2012 SARS epidemics, which started in Asia and spread worldwide. This virus causes the characteristic respiratory illness, which includes high fever, dry cough, and difficult breathing, and severe cases manifested by respiratory failure and death (86). This SARS coronavirus emerged from the bat reservoir (87). It infected palm civets as an intermediate host and then jumped to human hosts (87). A total of 8096 infections happened across 26 countries, with a high mortality rate of ∼10%, causing a total of 774 deaths (88).

As it occurred 17 years ago, research communities have had enough time to investigate the immediate and long-term impacts of SARS-CoV on the human CNS. SARS-CoV was found to trigger many neurological abnormalities such as encephalitis, aortic ischemic stroke, and polyneuropathy (86). Interestingly, most SARS-CoV cases displayed signs of cerebral edema and meningeal vasodilation in autopsy studies. Within the brain, the presence of SARS-CoV viral particles and their RNA sequences were identified concomitantly with ischemic changes of neurons, demyelinating abnormalities, along with evidence of monocyte and lymphocyte infiltrations in the brain (89). Table 2 has been provided with a comparative percentage of patients displaying neurological disorders.

**TABLE 2:** Neurological Manifestations by Respiratory viruses.

Virus	Primary site of Pathology	% Patients with Neurological Manifestations
Human respiratory syncytial virus (hRSV)	Lungs	≈ 1.8 %
Influenza Virus (IV)	Lungs	spectrum upto 6–19.1%
Nipah Virus (NiV)	Lungs	≈ 20 %
SARS-CoV	Lungs	≈ 15–20 %
MERS-CoV	Lungs	≈ 20 %
SARS-CoV-2 (COVID-19)	Lungs	≈ from 36% upto 84%

## MERS-CoV

MERS-CoV is a relatively recent member of the coronavirus family, identified in 2015 in the Arabian Peninsula. It originated from bats and used camels as an intermediate host for many years before jumping to human hosts (90). It has similar clinical outcomes to SARS-CoV, with typical pneumonia-like symptoms such as high fever, cough, and dyspnea, which often leads to acute respiratory distress syndrome (ARDS), collectively termed Middle East Respiratory Syndrome (MERS) (90). Severe conditions also included septic shock because of cytokine storm resulting in multiple organ failure and eventual death (91). This virus is considered a potential neuroinvasive virus, based on clinical and experimental evidences. Studies reported that almost 25.7% patients of MERS developed insanity and around 9% patients were getting seizure attacks after the MERS-CoV infection (92–94). Another study also confirmed these trends and stated that almost 20% of patients showed neurological symptoms upon MERS-CoV infection along with their regular respiratory illness. These symptoms included loss of consciousness, ischemic strokes, Guillain-Barre syndrome, paralysis, and other infectious neuropathic manifestations.

## SARS-CoV-2/COVID-19

The latest addition of novel coronavirus, first observed in 2019 in Wuhan city of China, has been named SARS-CoV-2 based on its genome sequence analysis (95, 96). This novel coronavirus shares a 79.5% sequence similarity with earlier SARS-CoV and an almost 50% similarity with MERS-CoV. SARS-CoV-2 causes symptoms typical of pneumonic respiratory illnesses, such as varying degrees of fever, cough, and pneumonia, with an extensive risk of multiple organ failure with a ∼4% mortality rate observed so far (96).

ACE2 is an established receptor for many human coronaviruses and influenza virus (97, 98). Inside the CNS, binding of SARS-CoV-2 with this receptor may cause elevated blood pressure and cerebral hemorrhage. ACE2 is also expressed in the capillary endothelia of the CNS; SARS-CoV-2 could bind and break the blood-brain barrier to enter the CNS (99). Since SARS-CoV-2 shares almost an ∼80% genome sequence similarity with previous SARS coronavirus, we can expect a lot of similarities in various protein structures and their mode of pathogenesis as well.

## Neurological Anomalies Caused by SARS-CoV-2

Since the pandemic is still going on, long-term secondary effects of SARS-CoV-2 infection on patient’s health is yet to be observed by clinicians. Neurological abnormalities are still recorded at patient levels, and their molecular mechanisms and animal model studies and cellular data are yet to be performed and validated. Clinical reports have shown that SARS-CoV-2 infected patients display the symptoms of intracranial infection like headache, loss of consciousness, epileptic seizures, etc. A majority of patients reported loss of smell and taste, a condition known as anosmia and dysgeusia (100–102). Interestingly, the asymptomatic patients who did not display clear respiratory illnesses were also experiencing these neurological symptoms (103–105). Many Chinese hospitals have constantly reported that novel SARS-CoV-2 infection has attacked the CNS of patients and these patients have displayed symptoms of viral encephalitis (105). Genome sequencing data from the cerebrospinal fluid of SARS-CoV-2 infected patients have also detected the viral sequences, which added support to the idea that SARS-CoV-2 can invade the CNS and could likely be the cause of the neurological anomalies associated with the disease (103–105). However, a direct cause-and-effect relationship between SARS-CoV-2 and neurological abnormalities is yet to be established experimentally. It is plausible that many opportunistic pathogens might permeabilize the blood-brain barrier and cause secondary intracranial infections. This might be the cause of the above reported symptoms of headache, vomiting, limb convolutions, and consciousness disorders apparent in severe COVID-19 patients. COVID-19 is primarily a lung infection and causes a massive peripheral “cytokine storm,” which partially explains why this infection would also cause acute cerebrovascular disease (106, 107). Other patient reports also suggest that severe SARS-CoV-2 infections is often associated with elevated blood levels of D-dimers and significant platelet reductions, again giving some explanation as to why the patients are at a higher risk of cerebrovascular events in their body.

Human coronaviruses share a strong similarity with neuroinvasive animal coronaviruses, such as PHEV (porcine hemagglutinating encephalitis virus) (108), FCoV (feline coronavirus) (109), and JHM virus (110), regarding structure and mode of replication. All these animal coronaviruses can invade the CNS and cause multiple neuropathologies. Out of eight reported human coronaviruses, four of them, [HCoV-229E, HCoV-OC43 (21, 108), SARS-CoV (21, 36), and MERS-CoV (21, 36)] have been reported to be neuroinvasive and neurotropic in humans. This suggests that we would observe a heavy burden of neurological abnormalities in SARS-CoV-2 patients in the future. The SARS-CoV-2 pandemic is also more worrisome since it has already infected a very large amount of the human population globally (∼19,000,000), and is still ongoing.

## Possible Interventions and Defense Strategies Against SARS-CoV-2 and COVID-19

As the COVID-19 pandemic has crippled socio-economic activities and human interactions globally, the world needs urgent measures to contain and suppress the viral transmission as well as get an effective vaccine as a long-term goal. Based on available clinical and epidemiological data so far, the World Health Organization (WHO) has already issued many guidelines which has helped in designing the prevention strategies to suppress the general viral transmission and mortality rate due to SARS-CoV-2. A controlled movement of population, increased and rapid testing followed by isolation, and a wider availability of healthcare services to reduce mortality are a few commonly practiced suggestions which have controlled this disease to a great extent. Since neurological disturbances were more frequent in severely ill and critical patients of COVID-19 as compared to mild ones (104), this suggests that early diagnosis, lowering the viral load, and suppression of acute host inflammatory response is of paramount importance in combatting neurological disturbances. Since an uncontrolled cytokine storm has been generally implicated in multi-organ failure and worsening of neurological symptoms, the main focus during medical care of COVID-19 patients has shifted largely to mitigate the host’s inflammatory responses (111). Several candidate drugs such as Baricitinib, fedratinib, and ruxolitinib (anti-inflammatory drugs used for rheumatoid arthritis), Hydroxychloroquine, Azithromycin, Remdesivir (a combination used as anti-viral treatments), a combination of lopinavir and ritonavir (LPV/r), Corticosteroid for calming down cytokine storms, recombinant Interferons, and many other popular anti-cancer drugs have been tested and reported to show partial relief in combatting the overall disease severity [reviewed in detail by 111].

## Influenza Virus (IAV)

Influenza viruses are a member of the Orthomyxoviridae family and contain negative-sense single-strand RNA as their genome (112). There are four distinct types of Influenza viruses known so far: A, B, C, and Thogotovirus. However, only A and B type are clinically relevant for causing human diseases (112). The symptoms are characteristic of respiratory illnesses such as flu, and include chills, cold, headache, muscular body pains, and sore throat (113). Influenza causes widespread seasonal epidemics, affecting 3–5 million humans, of which 10% could turn lethal (114). It usually causes a localized infection of the upper respiratory tract, but in a few severe cases it can affect the lower tract and cause pneumonia-like symptoms (115–117) and even affect the CNS homeostasis (116, 117). It was reported that Influenza infection could act as an aggravating factor for Parkinson’s disease (PD) (118). Mouse experimental studies also suggested that Influenza virus can enhance ischemic brain injury by initiating a cytokine cascade and risk of cerebral hemorrhage via disrupting the tissue-type plasminogen activator (119).

In the case of Influenza infections, there is an extensive history of patients reporting a variety of CNS disorders which collectively suggested that Influenza virus is a potentially neurotropic virus and is capable of giving long-lasting neurological sequelae (116). Even some recent outbreaks and the 2009 “Swine Flu” pandemic also gave similar indications that neurological consequences are a much likely after-effect of Influenza infection (120, 121). Although being notorious for respiratory disease outcomes, their second most common disease manifestations are encephalitis and other CNS complications such as ataxia, myelopathy, seizures, and delirium, which usually appear after 1 week of respiratory symptoms of influenza (120, 121). Multiple studies have confirmed the neurological sequences of Influenza infections like encephalitis, febrile seizure, Reye’s syndrome, acute encephalomyelitis (ADEM), and acute necrotizing encephalopathy in humans (121). By using murine models, it has been established that Influenza A virus enters the CNS via the olfactory nerve route and disrupts the hippocampal morphology and expression levels of synaptic regulatory genes to alter the cognition and behavior of the host (122). It can also infect the microvascular endothelial cells and breaks the BBB to enter the CNS (123). However, in the case of Influenza infection, interestingly, just having the capacity to enter the CNS doesn’t translate into it being neuro-pathogenic (7). It was best exemplified during the 2009 pandemic caused by H1N1 influenza, which never entered the CNS but was well-documented to have a strong association with encephalitis (124).

However, this paradox can be explained from a neuroinflammation perspective. Peripheral “Cytokine storm” caused by viral infections can explain why being neurotropic is not an essential criteria for neurological abnormalities. The peripheral cytokines can travel to the CNS and indirectly activate or prime the brain resident microglia (as explained in the above sections) (125, 126). In the case of the non-neurotropic H1N1 strain (A/PR/8/34) infection, which couldn’t enter the CNS but produced a massive central inflammatory response, altered the hippocampus, and caused subsequent cognitive deficits in mice (126). In this study, they checked the hippocampal status just 7 days post infections, however, an intriguing question is left regarding the long-term consequences of influenza-related neuropathology. Another study by (127) also provided convincing experimental evidence that common Influenza (non-neurotropic strain) can alter the hippocampus and influence the behavior of patients even after infection was reclined (127).

## Nipah Virus (NiV)

Nipah virus (NiV) is one of the most recent emerging members of the paramyxoviridae family and causes acute respiratory illness in human hosts (128). In critical cases, it is also reported to manifest necrotizing alveolitis, hemorrhage, pneumonia, and pulmonary edema (129). It first originated in Malaysia during 1998–1999 and has a very high mortality rate, up to 40% (130). Immunohistological studies of fatal patients confirmed the widespread presence of viral particles and other NiV antigens from endothelia and smooth muscle cells of blood vasculature (131). Parenchymal cells, like muscle cells and even neurons, were found to be abundantly infected with NiV (131). Particularly, blood endothelial cells and neurons are suggested as the main site of infection in the CNS and vasculitis and thrombosis were considered critical for NiV pathogenesis (131, 132). Mouse model studies have confirmed that NiV takes the olfactory nerve route from nasal epithelia to enter the CNS (133). NiV is reported to cause many neurological pathologies such as confusion, motor deficits, reduced consciousness, seizures, and febrile encephalitic syndrome (134).

## Measles Virus (MV)

Measles virus (MV) is a common name for genus Morbillivirus, belonging to the subfamily Paramyxovirinae of the family Paramyxoviridae. MV falls in the category of an enveloped virus with a genome of a single-strand, negative sense RNA (135). Measles virus is a very common virus that causes a respiratory disease with fever, cold, and congestions (135).

MVs are speculated to enter the CNS by both routes: the hematogenous and neuronal transport pathways. However, there is evidence showing both kinds of route taken by the MV virus to reach the CNS. MV nucleocapsid protein has been consistently reported at the pre-synaptic membranes of infected neurons, which suggest that MV might spread in a contact dependent, *trans-*synaptic fashion. The paradox of their traveling route has been reviewed in detail by Young et al. (136, 137). It is mostly a self-limiting disease, however, many severe patients are reported to have post-infectious encephalomyelitis (PIE) or acute disseminated encephalomyelitis (ADEM) (136). In few immunocompromised patients, MV infection can lead to Measles inclusion body encephalitis (MIBE), which is a secondary CNS complication (138). Another form of CNS disease, Subacute Sclerosis Pan Encephalitis (SSPE), has also been associated with some critical MV patients. It is regarded as a slow-progressing neurological disease that only surfaces 6–10 years post MV infection (135–137).

## Conclusion, Future Directions, and New Insights

In this review article, we have discussed how respiratory viruses take advantage of olfactory nerve cells and sometimes even the hematogenous route to reach the CNS. Bystander damage to multiple organs such as the brain, kidney, and heart takes place, although the lungs remain its major battlefield. Peripheral cytokine storms, elevated hypoxia injury, and inflammatory activation of CNS cells like microglia play key roles in causing neurological damage to patients infected with respiratory viruses. This review article presents the compilation of previously known interrelations of respiratory viruses and disruption of CNS homeostasis. Specifically, all the previous members of the coronavirus family have been shown to impair neurological health as a long-term sequelae. SARS-CoV-2 has already infected over 19 million people around the globe and killed more than 700,000 people globally. In the future, a very large number of SARS-CoV-2 patients are likely to experience a myriad of neurological abnormalities.

There should be a global effort toward not only epidemiological mitigation measures, like controlling mass people movements, cross-continental travel, and developing effective vaccines, but also gathering more data about neurotropism, the disruption of glial cell biology, and the role of microglia in amplifying the cytokine storm by SARS-CoV-2. Our previous works have specifically elucidated how a virus infection (HIV-1, DENV, JEV, etc.) or even viral toxic proteins can cross the blood-brain barrier and aggravate the neuroinflammatory response within the CNS. We have also highlighted in our previous work how extracellular vesicles released by peripheral immune cells after Dengue virus infection have the capacity to trigger a cytokine storm phenomenon in the CNS. These recent works provide us with more clues about how we can block the interconnections of periphery cells and the CNS. We can further explore the trafficking biology to control the extracellular vesicle secretion which transfers the signals and transcriptional activators between organs. We can also explore various temporary measure to regulate BBB permeability, so that SARS-CoV-2 crossing over to CNS could be checked. Enhancers for mucosal immunity should be explored, which can contain SARS-CoV-2 at epithelial surfaces itself and not allow them to invade all the vital organs of the host, including the CNS.

This awareness would offer us an opportunity to adopt a much more holistic approach of neurological healthcare management while the acute phase of the SARS-Cov-2 is still ongoing, in order to improve the upcoming life-quality of SARS-CoV-2 and other respiratory patients.

## Author Contributions

RM and AB conceptualized the framework of this review article, corrected, read, and finalized the article. All authors contributed to the article and approved the submitted version.

## Conflict of Interest

The authors declare that the research was conducted in the absence of any commercial or financial relationships that could be construed as a potential conflict of interest.
